# Editorial: Emerging researchers in frontiers in pharmacology: obstetric and pediatric pharmacology 2022

**DOI:** 10.3389/fphar.2023.1215954

**Published:** 2023-05-25

**Authors:** Qiwei Yang, Katia Candido Carvalho, Reza Shirazi, Ali Falahati

**Affiliations:** ^1^ Department of Obstetrics and Gynecology, University of Chicago, Chicago, IL, United States; ^2^ Clinical Hospital, Faculty of Medicine, University of Sao Paulo, Sao Paulo, Brazil; ^3^ Department of Anatomy, School of Medical Sciences, Medicine, UNSW Sydney, Sydney, NSW, Australia; ^4^ Department of Biology, Yazd University, Yazd, Iran

**Keywords:** risk factors, pregnancy complication, infertility, uterine fibroids, ovary diseases, reproductive diseases, exosomes, maternal-fetal pharmacology

## 1 The aim and scope of this Research Topic

Globally, while students are undertaking fundamental research as part of their education in Obstetric and Pediatric Pharmacology, most of this research needs to be conveyed to the broader audience. We acknowledge that many student researchers may find peer review daunting. Therefore, at Frontiers, where peer review is considered a collaborative process, our interactive peer review is tailored to provide researchers with hands-on guidance and constructive feedback. This specific Research Topic is to encourage emerging researchers to publish their work with Frontiers in Pharmacology. In addition, our Research Topic Editors are committed to advancing emerging researchers’ and students’ success at publications.

This Research Topic features 12 articles, including 5 original and 7 review articles, in a multidisciplinary collaboration among obstetrics and gynecology, pharmacology, and pediatrics. These articles cover several diseases, including pregnancy complications, children’s diseases, uterine fibroids, and ovarian disorders.

## 2 Overview of contributors

### 2.1 Diseases during pregnancy

Adverse pregnancy outcomes are known to have long-term health implications for the child. Fetal arrhythmias are common cardiac abnormalities associated with high mortality by reason of ventricular dysfunction and heart failure (Keenan et al., 2022). Qin et al. conducted a network meta-analysis to understand the efficacy and safety of various therapeutic medicines for fetal tachycardias, one of the fetal arrhythmia types. The authors concluded that flecainide monotherapy and a combination of digoxin and flecainide should be the superior therapeutic strategies for fetal tachycardia.

Ectopic pregnancy (EP) has the implantation of a fertilized ovum outside the endometrial cavity (Barnhart, 2009), most often occurring in a fallopian tube. Predictive factors of damage to the Fallopian tube may guide the treatment for patients with tubal pregnancy (Cabar et al., 2006). Some potential predictors of trophoblastic invasion of the fallopian tubes have been reported in EP. Teshima et al. investigated the link between VEGF tissue expression and the depth of trophoblastic infiltration into the tubal wall in patients with ampullary pregnancy, which is not associated with the previous finding that serum VEGF was correlated with the trophoblast invasion in the tubal wall from EP patients. This study indicates that the tissue expression of VEGF at the EP implantation site may not be primarily responsible for providing the local microenvironment triggering the trophoblast invasion.

The use of prescription drugs is prevalent during pregnancy, Pregnant women can have severe health consequences for infants if misuse of prescription drugs occurs. Currently, the knowledge about maternal-fetal safety and efficacy of drug use is limited. Hudson et al. present an overview of the current understanding of maternal-fetal drug exposure, discuss biospecimen-guided sampling design and methods for measuring fetal drug concentrations throughout gestation, and propose strategies for advancing pharmacology research in the maternal-fetal population.

Some women experience health problems during and after pregnancy. Pregnant individuals with arterial hypertension have significantly high risks of maternal mortality (Vaidy and Vaidya, 2023). Brandão et al. reported that assessment by ambulatory blood pressure monitoring (ABPM) verified the presence of hypertension in pregnant women. The ethnicity, self-reported hypertension, and the presence of hypertension during pregnancy are associated with arterial hypertension measured by ABPM. Measurement of arterial hypertension by ABPM will help improve quality of life and longevity.

Antiphospholipid syndrome (APS) is an autoimmune disorder that causes an increased risk of blood clots (Garcia and Erkan, 2018). As a result, pregnant women with APS show an increased risk of miscarriage. Wu et al. conducted a bibliometric analysis to review the studies in the field of APS and revealed that the research on APS has increased steadily in the past 10 years. Clinical studies on the mechanism and treatment of APS are recognized as encouraging research hotspots to reduce APS-associated miscarriages.

Preeclampsia complicates 2%–4% of all pregnancies and accounts for about 46,000 maternal deaths and 500,000 fetal or newborn deaths yearly (Magee et al., 2022). In the review article by Veiga et al., some inflammatory markers, including leptin, total cholesterol, triglycerides, C-reactive proteins, and TNFa, were elevated in pregnant women with preeclampsia compared to pregnant control women, indicating the correlation between the inappropriate inflammatory responses and preeclampsia pathophysiology.

### 2.2 Children diseases

Apnea of prematurity is a developmental disorder affecting most highly preterm infants and associates with long-term morbidity, including poor neurodevelopmental outcomes (Williamson et al., 2021). Caffeine has been used for many years to treat apnea of prematurity (Chavez and Bancalari, 2022). However, the long-term use of caffeine may cause adverse effects. In the review article by Dai et al., caffeine alters the circadian rhythms in humans and animals, and the relationship between preterm infants and circadian rhythms linked to caffeine therapy could help in the clinical practice to encourage precision therapy. Further investigation into the effect of caffeine on circadian rhythms regarding safety, dose efficacy, and duration of treatment during pregnancy is needed. In addition, obstructive sleep apnea hypopnea syndrome (OSAHS) is a sleep-related breathing disorder associated with substantial morbidity. A clinical trial by Zheng et al. tested the safety and efficacy of esketamine during drug-induced sleep endoscopy (DISE) in children with OSAHS, and compared it with dexmedetomidine, a selective alpha-2 adrenergic agonist, and recommended agent for DISE. Their studies demonstrated that esketamine provided a more effective and safer depth of anesthesia for pediatric DISE with OSAHS than dexmedetomidine.

Children experience severe repercussions from poisoning due to less capability of neutralizing harmful substances. In addition, developmental exposure to adverse exposure can increase the risk of diseases in the adult stage. Althobaiti et al. performed a retrospective cohort study on 122 children exposed to various toxic substances in Makkah, Saudi Arabia, including pharmaceutic products, household products, plant envenomation, and animal envenomation. They identified the poison forms, poisoning routes, and presenting symptoms. Their studies indicate that acute poising among children is a significant health Research Topic that necessitates more attention to raise awareness of safety requirements.

### 2.3 Uterine and ovarian diseases

Uterine fibroids (UFs) are the most common pelvic tumors among women of reproductive age, affecting more than 75% of women. Although benign, UFs are associated with significant morbidity, including heavy menstrual bleeding, pelvic pain, and reproductive dysfunction. UFs are the leading cause of hysterectomy (Bulun, 2013; Yang et al., 2022). Women of African descent are at a higher risk of developing UFs and frequently experience much more severe symptoms (Li et al., 2023). Sub-Saharan Africa is known to have the largest population of black women. However, most UFs studies do not include people from the continent of Africa. Sefah et al. reviewed the existing literature, emphasizing that the prevalence of UFs on Africa is not well investigated. Therefore, conducting future research on African women is highly needed.

Epithelial ovarian cancer (EOC) is the most common type of ovarian cancer that affects the female reproductive system and continues as a leading cause of death from gynecological malignancies (Qu et al., 2022). Baghban et al. reviewed the studies on the role of exosomes in EOC. They revealed that research on the exosome and EOC had been expanded, and China is much more involved than other countries in research, financial support, and international cooperation. The interest of exosome-oriented research on EOC includes exosomes as prognostic and diagnostic biomarkers, the role of exosomes in proliferation, migration, and metastasis, epithelial-mesenchymal transition, and chemoresistance. In this Research Topic, another review article by Izadi et al. presented an overview of mesenchymal stem cells (MSC)-derived exosomes as a promising approach for treating infertility in women with polycystic ovary syndrome (PCOS). MSC-derived exosomes exhibited therapeutic effects on the PCOS via modulating immunity response, exerting an anti-inflammatory effect, and suppressing apoptosis of granulosa cells. These two reviewer articles highlight and suggest the promising role of exosomes in targeting ovary diseases, including PCOS and ovarian cancer.

## 3 Conclusion

In conclusion, this Research Topic has provided original research and updated reviews of early-stage researchers related to basic and translational research in obstetrics and gynecology, pharmacology, and pediatrics. These studies further advance our understanding of the risk and pathogenesis of infertility, adverse pregnancy outcomes, uterine fibroids, and ovarian diseases. The evidence collected from this Research Topic is also expected to be translated into more precise and practical clinical approaches to predict and treat relevant human disorders in the future.
